# Efficacy and visual outcomes of the foldable capsular buckle scleral buckling in rhegmatogenous retinal detachment

**DOI:** 10.3389/fmed.2024.1412048

**Published:** 2024-07-29

**Authors:** Guohua Jiang, Yuan Lin, Yan Chen, Huping Wu

**Affiliations:** ^1^Xiamen Eye Center and Eye Institute of Xiamen University, Xiamen, China; ^2^Xiamen Clinical Research Center for Eye Diseases, Xiamen, Fujian, China; ^3^Xiamen Key Laboratory of Ophthalmology, Xiamen, Fujian, China; ^4^Fujian Key Laboratory of Corneal and Ocular Surface Diseases, Xiamen, Fujian, China; ^5^Xiamen Key Laboratory of Corneal and Ocular Surface Diseases, Xiamen, Fujian, China; ^6^Translational Medicine Institute of Xiamen Eye Center of Xiamen University, Xiamen, Fujian, China

**Keywords:** foldable capsular buckle, scleral buckling, visual outcomes, rhegmatogenous retinal detachment, retinal detachment

## Abstract

**Objective:**

To investigate the difference in the effectiveness and refraction of the foldable capsular buckle (FCB) in rhegmatogenous retinal detachment (RRD).

**Methods:**

Six patients with simple RRD were treated for FCB scleral buckling at Xiamen Eye Center of Xiamen University from October 2023 to February 2024. The parameters assessed included demographic data, clinical data such as preoperative ocular axis, corneal endothelial count, macular foveal thickness, operative time, preoperative and final follow-up intro ocular pressure (IOP), retinal attachment status, and postoperative complications. Refractive change before and after surgery, including sphere, cylinder degree, spherical equivalent, and absolute spherical equivalent difference were compared.

**Results:**

All six patients with sound retinal reattachment after FCB scleral buckling, including two men and four women, mean age 41.33 ± 12.40 years old, duration before surgery onset to 7.17 ± 7.16 days, FCB mean operation time 36.67 ± 13.07 min, Preoperative IOP mean 13.35 ± 2.64 mmHg and mean 21.12 ± 8.09 mmHg of final follow-up IOP; there was no significant difference between preoperative IOP and follow-up IOP (*p* = 0.050). The preoperative sphere range was −6.25 to +2.50 D, and the cylinder range was −2.50 to +1.00 D; the absolute spherical equivalent difference before and after was 1.60 ± 1.69 degrees.

**Conclusion:**

FCB can achieve retinal reattachment and restore visual function in cases of RRD. The shorter duration of external scleral buckle compression with FCB suggests that FCB scleral buckling holds greater promise in the clinical treatment of RRD caused by retinal tears.

## Introduction

Rhegmatogenous retinal detachment (RRD) is the most common form of retinal detachment (RD), characterized by the separation between the neurosensory retina and the underlying retinal pigment epithelium and often induced by vitreous traction (1). This condition leads to fluid influx from the vitreous cavity into the subretinal space, ideally suitable for repair through a single surgical intervention (2). Scleral buckling (SB) surgery is widely employed as a therapeutic strategy in appropriate cases. However, it may pose challenges due to its steep learning curve and the requirement for meticulous preoperative examination and intraoperative retinal inspection (3). However, it yields better postoperative functional outcomes in cases of localized RRD and single retinal breaks, particularly in younger patients who have lenses (4).

The foldable capsular vitreous body (FCVB) consists of structures such as an inflatable balloon, drainage valve, and drainage tube, exhibiting excellent mechanical properties and biocompatibility (5). FCVB has been clinically validated as a safe and effective substitute for vitreous contents in supporting ocular structures in cases of severe retinal detachment or eyes reliant on silicone oil (6–8). Moreover, FCVB serves as a sustained-release system for intraocular medications (9, 10). Indications for FCVB implantation encompass a spectrum of ocular conditions, including complex and uncomplicated RDs (11). In RRD, FCVB called the foldable capsular buckle (FCB), serves as an external scleral tamponade. Clinical evidence supports the efficacy and safety of FCB scleral buckling in treating RRDs associated with simple, single-hole, non-proliferative vitreoretinopathy (12).

However, the innovative therapeutic approach of utilizing FCB as a novel scleral tamponade needs more comprehensive investigation regarding intraoperative procedures, postoperative outcomes, and visual acuity changes. Therefore, we conducted a prospective analysis of six cases of RRD treated with FCB scleral buckling surgery in our institution from 2023 to 2024 to observe the refractive changes in the eyeball following uncomplicated RRD.

## Methods

The research followed the ethical principles outlined in the Declaration of Helsinki. The Human Ethics Committee of Xiamen University, affiliated with Xiamen Eye Center, reviewed and approved the studies involving human participants. All patients provided written informed consent before surgery, including their agreement to use their data in future teaching and research at the institution.

### Patients

This prospective study enrolled six patients (six eyes) who underwent FCB scleral buckling surgery at the Xiamen Eye Institute from October 2023 to February 2024. A comprehensive analysis of electronic medical records for each patient was conducted, encompassing initial presentation details, age, gender, symptoms, diagnosis, treatment, complications, and visual outcomes.

The inclusion criteria included the absence of prior retinal surgeries, the presence of only one retinal break causing retinal detachment, retinal detachment involving no more than two quadrants, and a duration of detachment not exceeding 3 weeks. Exclusion criteria comprised known silicone allergy, keloid-prone individuals, intraocular inflammation, uveitis, a history of intraocular surgery in the contralateral eye, and severe systemic illnesses. Additionally, cases with retinal breaks larger than three disc diameters or involving multiple quadrants were excluded.

### Observational indicators

All patients underwent comprehensive ophthalmic examinations, including slit-lamp biomicroscopy, B-scan ultrasonography, optical coherence tomography of the retina, corneal endothelial cell count, and intraocular pressure measurement. Visual acuity was typically recorded using Snellen visual acuity charts and converted to LogMAR for analysis (13). For counting fingers or worse cases, the following conversions were applied: counting fingers, 2.0 LogMAR; hand motion, 2.3 LogMAR; light perception, 2.6 LogMAR; no light perception, 2.9 LogMAR. Additionally, surgical duration and postoperative retinal reattachment status, including the condition of the cornea and retina during follow-up, as well as the positioning of FCVB, were recorded. The postoperative spherical equivalent was measured 3 months after surgery, which we consider to be the time when visual recovery reaches its optimal level.

### Surgical method

Two experienced physicians, Guohua Jiang, and Yan Chen, performed all surgeries collaboratively at the Xiamen Eye Center and Eye Institute of Xiamen University. The foldable capsular vitreous body (FCVB) AV-10P model (Vesber, Guangzhou, China) was utilized in the FCB group. It comprises silicone rubber, an inflatable balloon, a drainage valve, and a drainage tube. Balanced salt solution (BSS) can be injected into the capsule membrane via the tube-valve system to exert pressure on the sclera (14).

After administering local anesthesia, a small incision was made in the conjunctiva using a minimally invasive technique. A folded FCB was inserted through a tri-folded balloon. Tenon’s capsule was dissected to form a tunnel, and subretinal fluid was either drained or retained as needed. The FCB was then implanted along the pre-formed tunnel, with its convex surface facing the sclera and positioned over the retinal break. The drainage tube was secured to the sclera, and an appropriate amount of physiological saline was injected through the drainage valve. The fundus was examined to confirm the FCB’s positioning over the retinal break and to verify retinal reattachment. Finally, the drainage valve was covered and the conjunctiva and Tenon’s capsule were sutured (14). Additionally, no cryotherapy was performed during the surgical process.

The average duration for postoperative laser surgery typically ranges from 3 to 10 days after the initial procedure. During this period, fluid absorption is confirmed through an indirect ophthalmoscope, and then laser treatment is applied to enhance and solidify the therapeutic effect. The BSS was removed approximately 2–4 weeks later.

## Results

Six patients, two men, four women, mean age 35.67 ± 15.99 years, onset day 6.67 ± 7.52 days, endothelial cell count 2314.80 ± 102.11 cells/mm^2^, preoperative macular foveal thickness 147.60 ± 21.75 μm, axial length of the affected eye was 27.52 ± 4.84 mm, and healthy eye axial length was 25.98 ± 1.63 mm. The duration of surgery time was 36.67 ± 13.07 min.

Preoperative IOP means 13.35 ± 2.64 mmHg and 21.12 ± 8.09 mmHg at the final follow-up; there was no significant difference between preoperative IOP and follow-up IOP (*p* = 0.050, one-way ANOVA test). Preoperative sphere range −6.25 to +2.50 D, preoperative cylinder range −2.50 to + 1.00 D; postoperative sphere range −7.50 to +2.50 D, preoperative cylinder range −2.50–0.00D. The absolute spherical equivalent difference before and after was 1.60 ± 1.69 degrees. The refractive results before and after FCB surgery are shown in Table 1.

**Table 1:** Refractive results before surgery and final follow-up (one-way ANOVA test).

	Sphere	Cylinder	Spherical equivalent	BCVA
Preoperative	−2.25 ± 3.22	−0.50 ± 1.18	−2.50 ± 3.75	0.75 ± 0.77
Postoperative	−4.04 ± 3.52	−0.95 ± 0.84	−4.35 ± 3.99	0.56 ± 0.46
*p*-value	0.380	0.458	0.427	0.623

BCVA, Best corrected visual acuity.

**Figure 1:**
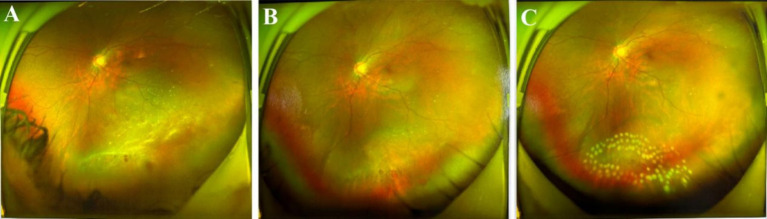
FCB aid retinal re-attachment. **(A)** Preoperative. **(B)** One day after surgery, the fundus image show reattached retina. **(C)** After the postoperative photocoagulation treatment. After personalized photocoagulation and subsequent adjuvant treatment, the holes were closed and the retina was well attached.

All patients received postoperative photocoagulation therapy (Figure 1). All patients had good retinal anatomical recovery during the postoperative follow-up period. No significant adverse events occurred during the follow-up period, including infection, endophthalmitis, cardiovascular events, and other systemic reactions. The shortest time for liquid removal in the balloon was 16 days. Diplopia appeared in all patients 2–4 weeks before the removal of the FCB, and the symptoms disappeared immediately after removal. Extraocular muscle movement (EOM) limitation occurred in two cases when the FCB was fixed under the rectus muscle, but the degree of EOM was generally tolerable for the patients (Table 2). At 3 weeks after surgery, one FCB patient had a balloon displaced, and no other complications occurred after adjustment; none of the patients experienced severe postoperative bleeding or intense discomfort.

**Table 2:** Patient background, treatment, and visual outcome.

	Sex	Age	eye	Operation time, minutes	Preoperative IOP, mmHg	3 months IOP, mmHg	Sphere	Cylinder	Spherical equivalent	Preoperative BCVA	3 months sphere	3 months cylinder	3 months spherical equivalent	3 months postoperatively, logmar	Total spherical equivalent difference	Complication
Eye 1	M	14	OS	30	17.1	32.5	−4.75	−1	−5.25	0.22	−7.5	−0.75	−7.875	0.39	−2.52	Too much BBS, EOM limitation
Eye 2	M	40	OS	34	10.9	17.7	−1.5	−0.5	−1.75	2	−6	−1	−6.5	0.52	4.25	
Eye 3	F	31	OD	30	16.3	29.5	−6.25	−2.5	−7.5	0	−6.5	−2.5	−7.75	0	0.25	EOM limitation
Eye 4	F	55	OS	55	11.9	11.5	0	0	0	1.39	0	0	0	1.39	0	
Eye 5	F	23	OS	21	11.6	18.5	−3.5	0	−3.5	0.39	−5	−0.5	−5.25	0.39	1.75	
Eye 6	M	51	OD	50	12.3	17	2.5	1	3	0.52	0.75	−1	1.25	0.69	1.75	FCB displaced

M, male; F, female; BCVA, Best corrected visual acuity; OD, right eye; OS, left eye; ECM, extraocular muscle movement.

## Discussion

FCB is a high-purity medical silicone product suitable for long-term tamponade, verified by *in vivo* experiments and widely used (15–17). Previous studies have shown that FCB scleral buckling may be used as an alternative treatment for certain cases of complex RRD who refuse PPV surgery (14). Therefore, this study investigated buckling efficacy, safety, and refractive results using the modified FCB as a scleral buckle material. We believe that the buckling of FCB avoids the classic SB pull and more significant trauma. FCB enables faster implantation and easy adjustment, and the refractive results at the final follow-up can be confirmed.

**Figure 2:**
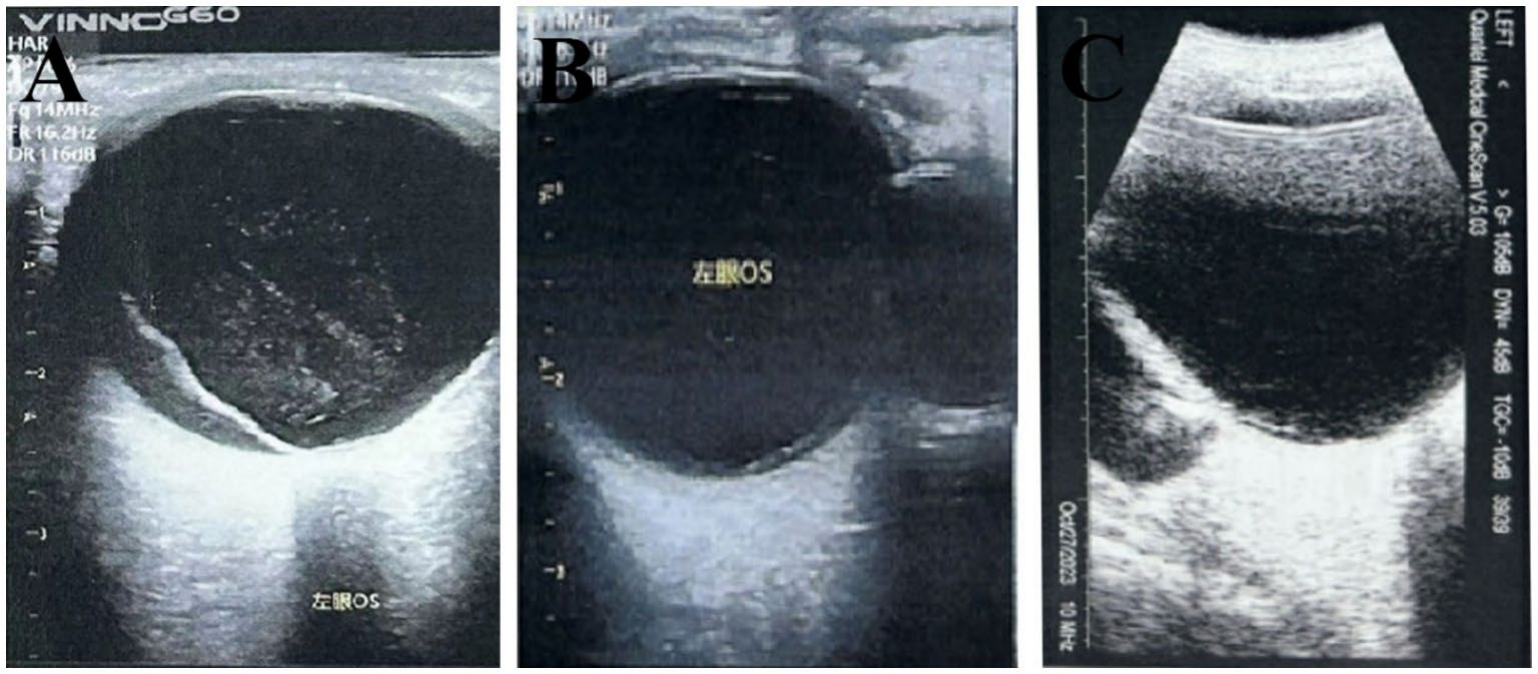
Top pressure state of the FCB in the RRD. **(A)** Preoperative. **(B)** The hole was closed 1 day after surgery, and the centum pressure was in place. **(C)** In postoperative follow-up, there was a stable balloon and no significant vitreous damage in the posterior segment of the eye.

Classical SB is considered an appropriate treatment for RRD, especially in simple cases with phakic and pseudophakic eyes (18). However, the operation of SB may lead to trauma and complications to the eyeball, such as macular hole formation, epiretinal membrane, residual subretinal fluid, proliferative retinopathy, elevated IOP, and EOM (19, 20). Postoperative infection, bleeding, diplopia, foreign body sensation, and recurrent subconjunctival hemorrhage caused by external pad material can also occur (21). This study observed the FCB can create a bulge by pressing on the sclera and seal the hole without disrupting the original vitreous body (Figure 2). Since FCB implantation does not require deep scleral fixation, it reduces the risk of scleral perforation and infection. In this study, the operation time for FCB resulted in a significantly shorter duration compared to traditional SB surgery, thereby reducing surgical trauma to periocular tissue and minimizing postoperative swelling and discomfort.

SB is a straightforward and effective technique for RD repair, especially for cases such as retinal dialyzes, round retinal holes, selected cases of RD associated with horseshoe retinal tears and as an adjunct to vitrectomy (22). Pressing the sclera through a scleral buckle offers advantages over other surgical approaches in treating RRD (23). Our study demonstrated that FCB could significantly improve the postoperative BCVA of patients during follow-up, and prevented retinal redetachment. However, a prospective study with a larger sample size and longer follow-up is needed to assess long-term visual acuity and refractive changes.

The prediction of poor visual outcomes after 12 months relies on repeated postoperative retinal redetachment and visual field loss within 7 days (24). Successful scleral buckling relies on the principle that retinal reattachment not only requires sealing the break but also relieving associated traction, which can enhance adhesion of the retinal pigment epithelium and choroid (25). Due to variations in patient holes and fundus conditions in RDD, FCB needs to personalize the management strategy in treating RDD. Therefore, our experience with FCB scleral buckling suggests making a conjunctival incision to insert the folded balloon, adjusting the amount of injected fluid (0.9–2.3 mL) based on intraocular pressure, and performing laser sealing of holes according to subretinal fluid absorption. In addition, whether all the holes are closed or secondary holes should be observed. According to the results of the indirect ophthalmoscopy, ocular ultrasound, and wide-angle fundus photography should be evaluated. If there is any omission, the FCB can be repositioned.

The limitation of this study is the lack of collection of postoperative ocular biological markers of patients for comparative preoperative and postoperative analysis. Furthermore, data were collected from a tertiary referral center, limiting the volume and potentially the comprehensiveness of variables assessed. Therefore, the study may differ from the data found in other settings. At present, we still need to conduct a multi-center study with more samples and follow-up time to confirm the safety and effectiveness of FCB in managing RDD.

## Conclusion

Based on our results, FCB scleral buckling surgery as a promising treatment method for treating RDD in clinical practice. FCB is characterized by its simplicity, safe, minimal tissue trauma, and rapid visual recovery better than classic SB in these aspects. However, regardless of the technique employed for RD repair, the success of the procedure hinges on the localization and treatment of all retinal breaks.

## Data availability statement

The data analyzed in this study is subject to the following licenses/restrictions: The data presented in this study are included in the article. The data are not publicly available due to restrictions that apply to the availability of the data (e.g., privacy or ethical). Datasets from this study may be available upon request from the corresponding author and provided upon approval from the sponsor and in accordance with data privacy and ethical provisions. Requests to access these datasets should be directed to: yuanlin_huaxiaeye@foxmail.com.

## Ethics statement

The studies involving humans were approved by the Human Ethics Committee of Xiamen University, affiliated with Xiamen Eye Center. The studies were conducted in accordance with the local legislation and institutional requirements. The participants provided their written informed consent to participate in this study.

## Author contributions

GJ: Data curation, Investigation, Writing – original draft. YL: Formal analysis, Investigation, Software, Writing – original draft, Writing – review & editing. YC: Data curation, Resources, Writing – original draft. HW: Conceptualization, Project administration, Supervision, Writing – review & editing.
